# Evolution‐guided multiomics provide insights into the strengthening of bioactive flavone biosynthesis in medicinal pummelo

**DOI:** 10.1111/pbi.14058

**Published:** 2023-04-28

**Authors:** Weikang Zheng, Wang Zhang, Dahui Liu, Minqiang Yin, Xia Wang, Shouchuang Wang, Shuangqian Shen, Shengjun Liu, Yue Huang, Xinxin Li, Qian Zhao, Lu Yan, Yuantao Xu, Shiqi Yu, Bin Hu, Tao Yuan, Zhinan Mei, Lanping Guo, Jie Luo, Xiuxin Deng, Qiang Xu, Luqi Huang, Zhaocheng Ma

**Affiliations:** ^1^ National Key Laboratory for Germplasm Innovation and Utilization of Horticultural Crops Huazhong Agricultural University Wuhan China; ^2^ Key Laboratory of Traditional Chinese Medicine Resources and Chemistry of Hubei Province, School of Pharmacy Hubei University of Chinese Medicine Wuhan China; ^3^ College of Tropical Crops Hainan University Haikou China; ^4^ National Resource Center for Chinese Materia Medica China Academy of Chinese Medical Sciences Beijing China

**Keywords:** evolutionary, multiomics, bioactive flavone, huajuhong medicines, pummelo

## Abstract

Pummelo (*Citrus maxima* or *Citrus grandis*) is a basic species and an important type for breeding in Citrus. Pummelo is used not only for fresh consumption but also for medicinal purposes. However, the molecular basis of medicinal traits is unclear. Here, compared with wild citrus species/*Citrus*‐related genera, the content of 43 bioactive metabolites and their derivatives increased in the pummelo. Furthermore, we assembled the genome sequence of a variety for medicinal purposes with a long history, *Citrus maxima* ‘Huazhouyou‐tomentosa’ (HZY‐T), at the chromosome level with a genome size of 349.07 Mb. Comparative genomics showed that the expanded gene family in the pummelo genome was enriched in flavonoids‐, terpenoid‐, and phenylpropanoid biosynthesis. Using the metabolome and transcriptome of six developmental stages of HZY‐T and *Citrus maxima* ‘Huazhouyou‐smooth’ (HZY‐S) fruit peel, we generated the regulatory networks of bioactive metabolites and their derivatives. We identified a novel MYB transcription factor, CmtMYB108, as an important regulator of flavone pathways. Both mutations and expression of *CmtMYB108*, which targets the genes *PAL* (*phenylalanine ammonia‐lyase*) and *FNS* (*flavone synthase*), displayed differential expression between *Citrus*‐related genera, wild citrus species and pummelo species. This study provides insights into the evolution‐associated changes in bioactive metabolism during the origin process of pummelo.

## Introduction

**Figure 1:**
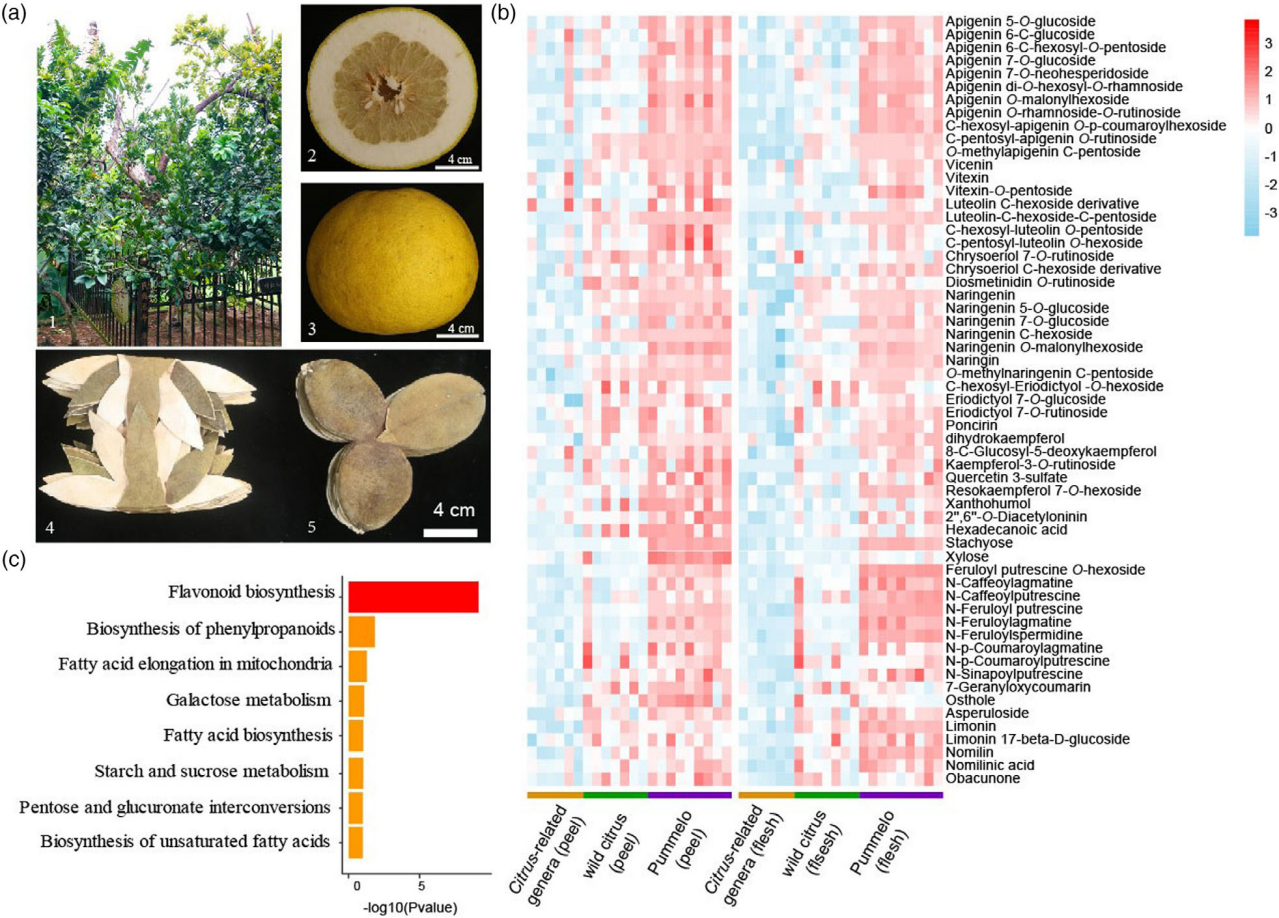
Characteristics of pummelo medicines and metabolic changes during the origin process of pummelo. (a) The 100‐year‐old HZY‐T king tree (1), equatorial (2) and shape (3) of HZY‐T mature fruit, seven claws (4) and three claws (5) Huajuhong medicines made from HZY‐T immature fruit peel, scar bar = 4 cm. (b) Heatmap of 59 metabolites that were higher levels in both flesh and peel of pummelo fruit compared with *Citrus*‐related or wild citrus species (*P* < 0.05, fold change >2). *Citrus*‐related genera (*n* = 6), wild citrus species (*n* = 7), pummelo (*n* = 9). Red represents high levels; sky blue represents low levels. (c) KEGG enrichment results of 59 metabolites with higher levels in pummelo.

In the Citrus genus, pummelo (*Citrus maxima* or *Citrus grandis*) is a basic species derived from the near northeastern India, northern Myanmar and northwestern Yunnan and more recently originated compared with *Citrus*‐related genera (such as *Atalantia*) and wild citrus species (such as *Citrus mangshanensis*) (Wang *et al*., 2017; Wu *et al*., 2018). Pummelo is used not only for fresh eating but also used as a medicinal plant for some landraces. The dried immature pummelo fruit peel, including all of flavedo and a small amount of albedo, was processed into traditional Chinese medicines, named Huajuhong that were one of the important treatment medicines of the COVID‐19 in China (Figure 1a) (Chinese Pharmacopoeia Commission, 2015). The medicines have efficient functions in reducing the risk of inflammatory diseases, oxidative stress, diabetes, dyslipidemia, endothelial dysfunction and atherosclerosis (Gualdani *et al*., 2016; Mahmoud *et al*., 2019; Zhao *et al*., 2021), due to the high abundance of flavanones, flavones and limonoids. Many studies have been shown that secondary metabolites were selected during the evolution and origin process of crops, such as carotenoids, sugar and cucurbitacin in watermelon (Guo *et al*., 2019), polyol/monosaccharide, cinnamyl alcohol and pectin in peach (Yu *et al*., 2018), and lignin and cellulose in coconut (Wang *et al*., 2021c). Despite the detailed origin of pummelo, the changes in the metabolome in the origin process of pummelo are largely unknown.

With the development of metabolome detection technologies, over 200 000 metabolites have been detected in medicinal plants, crops and model plants (Alseekh and Fernie, 2018; Rai *et al*., 2017). Subsequently, metabolome‐transcriptome association analysis (MTA) and mGWAS were used to find the regulatory network of metabolites (Chen *et al*., 2014; Li *et al*., 2020). The regulatory network of metabolites during the developmental stages of *Senna tora* (Kang *et al*., 2020), sweet orange (Feng *et al*., 2021) and kiwifruit (Wang *et al*., 2021b) have been described by MTA. Meanwhile, based on mGWAS, researchers have also identified many genes involved in the regulatory and synthesis of phenylpropanoids, flavonoids and terpenoids in Qingke, rice and tomato, respectively (Peng *et al*., 2017; Zeng *et al*., 2020; Zhu *et al*., 2018). Due to the limits of population numbers, the mGWAS has rarely been used to identify the regulatory network of bioactive metabolites in medicinal plants.

Bioactive metabolite synthetic pathways, such as the flavonoid pathway, phenylpropanoid pathway and terpenoid pathway, are usually regulated by MYBs, interacting with *chalcone synthase* (*CHS*), *chalcone isomerase* (*CHI*), *flavanone 3‐hydroxylase* (*F3H*), *flavonol synthase* (*FLS*), *cinnamate 4‐hydroxylase* (*C4H*), *squalene synthase* (*SQS*) and *oxidosqualene cyclases* (*OSCs*) (Liu *et al*., 2015; Zhang *et al*., 2020a). Some structural variations (SVs) and single nucleotide polymorphisms (SNPs) were generated in the promoters and body of MYBs during the evolutions or domestications of apple, strawberry and chilli pepper, which changed the expression and activity of MYBs and affected the levels of malic acid, anthocyanins and apsaicinoid metabolites, respectively (Castillejo *et al*., 2020; Jia *et al*., 2021; Zhu *et al*., 2019). However, the relationships between the changed genomic basis and bioactive metabolites accumulated in medicinal plants have been less studied.

In this study, we present a high‐quality reference genome of HuazhouYou‐ tomentosa (HZY‐T). Combining metabolome, genome and transcriptome analysis, we described the relationships between the genomic variation and metabolome changes during the origin process of pummelo, generated a bioactive metabolite regulatory network in HZYs fruit peel, and identified an important gene responsible for the high abundance flavonoids in pummelo fruit peel compared with *Citrus*‐related genera and wild citrus species.

## Results

### The metabolic profile of pummelo

To investigate the contribution of metabolites to the formation of medicinal‐purpose cultivars in pummelo and metabolic changes during the origin process of pummelo, we analysed metabolome of peel and flesh in six *Citrus*‐related genera, seven wild citrus species and nine pummelo species (Table S1). A total of 403 metabolites were detected in 22 accessions (Table S2). Principal component analysis (PCA) of metabolites divided 22 accessions into three groups, including *Citrus*‐related genera, wild citrus species and pummelo (Figure S1a,b). Compared with *Citrus*‐related genera or wild citrus species, 59 kinds of metabolites were at higher levels in both peel and flesh of pummelo. These metabolites included 2 kinds of carbohydrates, 1 kind of chalcone, 2 kinds of coumarins, 1 kind of flavanol, 11 kinds of flavanones, 21 kinds of flavones, 4 kinds of flavonols, 5 kinds of limonoids, 2 kinds of lipids, 9 kinds of phenamines and 1 kind of terpenoid (Figure 1b, Table S2). KEGG enrichment analysis revealed that metabolites with higher levels in pummelo (MHLPs) were mainly enriched in flavonoid biosynthesis and biosynthesis of phenylpropanoids (Figure 1c).

Of these MHLPs, 21/59 metabolites have anti‐inflammatory, anticancer and anti‐oxidative function to varying degrees and others, which were identified as bioactive metabolites; 22/59 metabolites were bioactive metabolite derivatives (Chen *et al*., 2016; Fan *et al*., 2019; Nasiri *et al*., 2021; Salehi *et al*., 2019) (Table S2). Among the bioactive metabolites and their derivatives, there are 18 kinds of flavones, 11 kinds of flavanones, 5 kinds of limonoids, 4 kinds of flavonols, 2 kinds of coumarins, one kind of chalcone, one kind of flavanol and one kind of terpenoid, such as vitexin, apigenin 7‐*O*‐neohesperidoside, naringin, naringenin 7‐*O*‐glucoside, limonin, nomilin, 7‐geranyloxycoumarin, obacunone and xanthohumol, which is consistent with the metabolic basis of Huajuhong medicines. Most bioactive metabolites and their derivatives were flavones and flavanones. Therefore, two flavones (vitexin and apigenin 7‐*O*‐neohesperidoside) and one flavanone (naringin) were analysed for their anti‐inflammatory function. The results indicated that vitexin, naringin and apigenin 7‐*O*‐neohesperidoside inhibited the expression of the proinflammatory cytokines (*COX‐2* and *IL‐6*) induced by lipopolysaccharide (LPS) *in vitro* and exhibited anti‐inflammatory activity (Figure S2a,b). Collectively, the formation of pummelo medicinal value was highly correlated with the origin of pummelo.

### Genomic characterization of a medicinal pummelo accession

**Figure 2:**
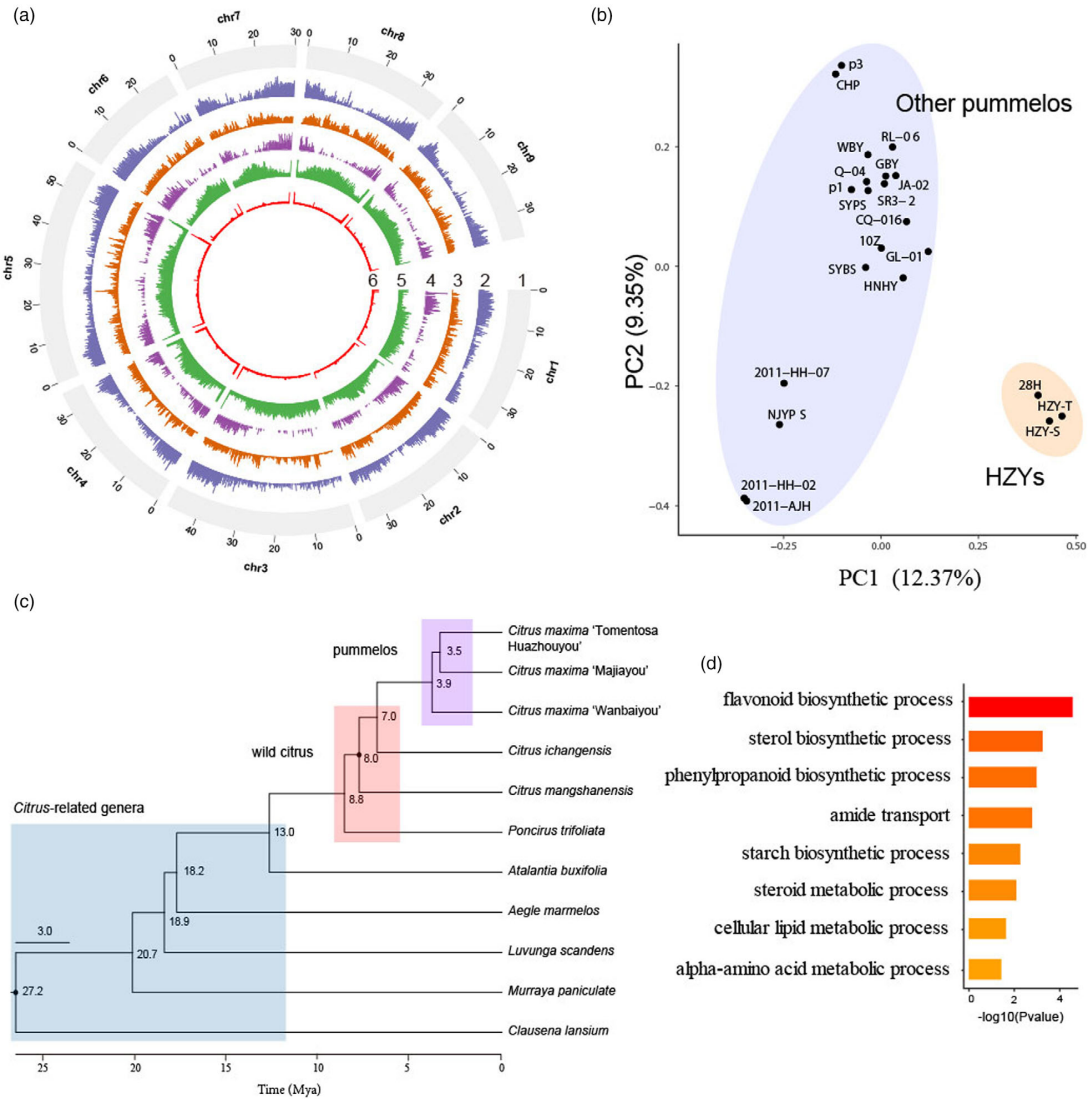
Genome features of HZY‐T pummelo. (a) Overview of the HZY‐T genome assembly. 1: Chromosomes, 2: Gene density, 3: SNP density, 4: density of SVs from 11 genomes, 5: TE density, 6: GC content. The SNPs and SVs were identified using the HZY‐T reference genome. (b) PCA of 22 pummelo accessions based on fourfold degenerate site SNPs. (c) Phylogenetic tree of citrus subfamilies based on orthologous genes. Mya, million years ago. (d) Gene Ontology enrichment analysis of genes that were expanded in pummelo compared with *Citrus*‐related genera or wild citrus species.

HZY‐T is one of the most important pummelo medicinal plants and is most widely processed in Hujuhong medicines. A 100‐year‐old HZY‐T tree, also named king tree, was found in Huazhou, Guangdong Province (Figure 1a). We *de novo* assembled a high‐quality genome of the tree king. The genome was sequenced using a combination of PacBio long reads from the PacBio Sequel platform, Illumina short reads and chromosome conformation capture (Hi‐C) technology. The assembled genome of HZY‐T is 349.07 Mb with a contig N50 of 1.74 Mb and nine chromosomes (Table S3, Figure 2a). To verify the quality of the assembly, we confirmed that 99.8% of the HZY‐T Illumina sequences could be mapped to the assembled genome. Assembly completeness was 99.1% by BUSCO assessment. Meanwhile, we ordered the assembled contigs and oriented them into nine pseudochromosomes using Hi‐C data (Figure S3). We annotated 26 924 genes for HZY‐T, which were distributed with an increase in density toward the ends of the pseudomolecules (Figure 2a). Meanwhile, we used a fourfold degenerate site at each SNP of 22 pummelo accessions (Table S4), including HZY‐T, HZY‐S and 20 published accessions (Wang *et al*., 2018), to perform the PCA analysis, which showed that pummelo accessions were divided into HZYs and other pummelo accessions (Figure 2b). The phylogenetic tree of pummelo based on the above SNPs also showed that the HZYs were grouped into one class (Figure S4).

HZY‐T and ten high‐quality genomes of Aurantioideae species from CPBD (http://citrus.hzau.edu.cn/), including *Clausena lansium*, *Murraya paniculate*, *Luvunga scandens*, *Aegle marmelos*, *Atalantia buxifolia*, *Poncirus trifoliata*, *Citrus mangshanensis*, *Citrus ichangensis*, *Citrus maxima* ‘Wanbaiyou’ and *Citrus maxima* ‘Majiayou’, were used to construct a phylogenetic tree and added to the time of fossil (Xie *et al*., 2013). The results reflected that these accessions diverged into three groups, *Citrus*‐related genera, wild citrus species and pummelo, and the *Citrus*‐related genera and wild citrus species had an earlier evolutionary origin than pummelo (Figure 2c), which is consistent with the conclusion previously reported (Wang *et al*., 2017). To investigate the genome changes during origin process of pummelo, we identified the 1405 expanded gene families from *Citrus*‐related genera/wild citrus species to pummelo (Table S5). These gene families were mainly enriched in flavonoids‐, sterol‐ and phenylpropanoid biosynthetic process (Table S6, Figure 2d). We also identified 7091 genes with SVs in pummelo compared with *Citrus*‐related genera/wild citrus species (Figure 2a, Table S7).

### Transcriptome analysis of genes correlated with flavonoids

**Figure 3:**
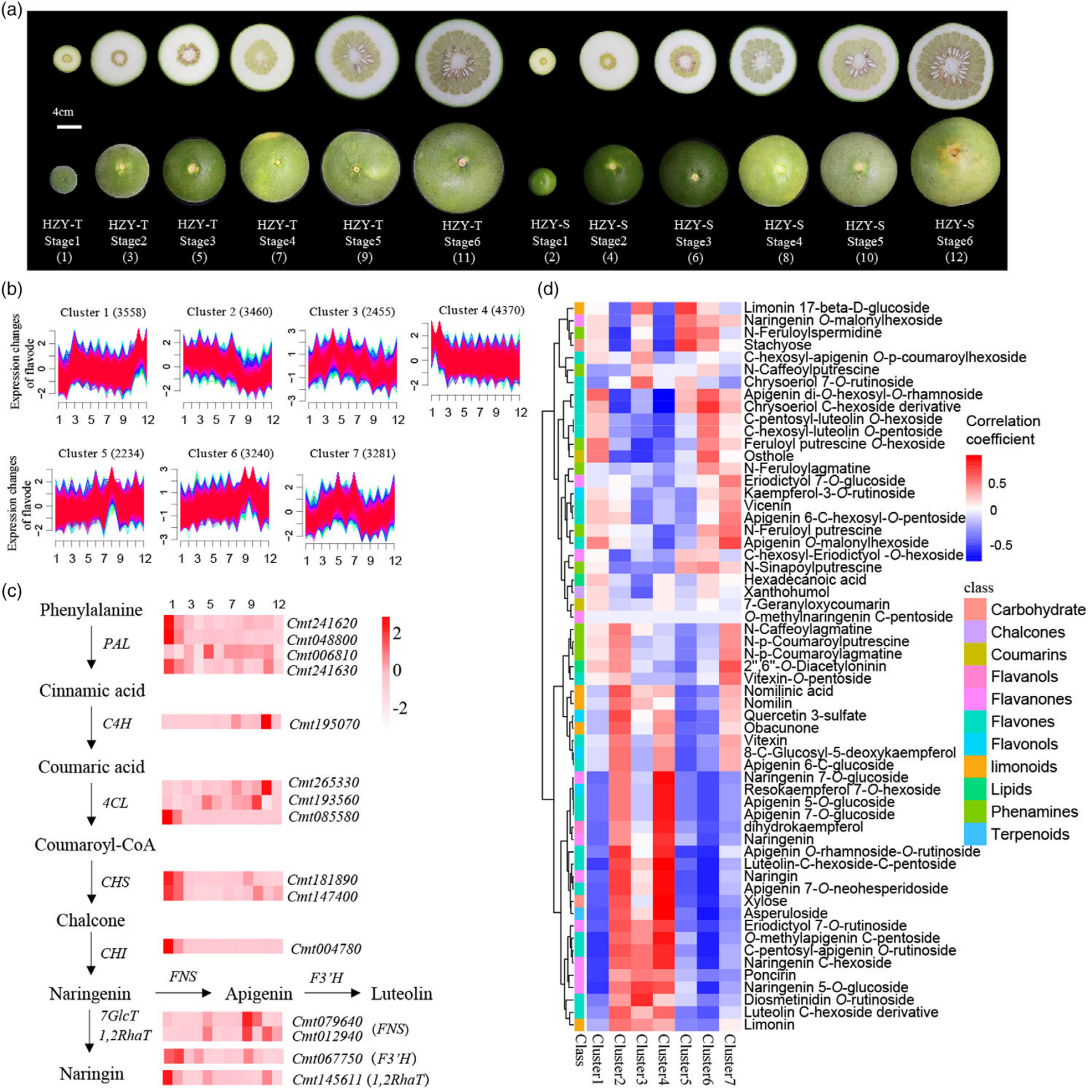
Dynamics of MHLPs and gene expression in different developmental stages of HZYs fruit flavedo. (a) Six developmental stages of HZY‐T and HZY‐S fruit. Bar, 4 cm. (b) Fuzzy c‐means clustering identified seven distinct temporal patterns of gene expression in the flavedo. 1, 3, 5, 7, 9 and 11 represent stages 1–6 of HZY‐T fruit, respectively. 2, 4, 6, 8, 10 and 12 represent stages 1–6 of HZY‐S fruit, respectively. The *y*‐axis represents log2‐transformed, normalized intensity ratios in each stage. (c) The relative expression of flavone and flavanone pathway genes. Red represents high expression, and white represents low expression. (d) Heatmap showing the correlation coefficient values between gene clusters and MHLPs in flavedo. Red indicates positive correlation; blue indicates negative correlation.

To investigate MHLPs transcriptional regulatory networks over the course of the HZYs fruit growth cycle, we collected two HZYs (HZY‐T and HZY‐S) flavedo and albedo of fruit peel in six developmental stages that were 45 DAF (days after flowering), 65 DAF, 85 DAF, 115 DAF, 145 DAF and 185 DAF, for a total of 24 samples (Figure 3a). Among these stages, the first three stages were usually considered the harvested and processed time of Huajuhong medicines. Analysis of 59 MHLPs in six different developmental stages of two HZYs fruit flavedo and albedo, showed that the accumulation of most bioactive metabolites and their derivatives were at higher levels in the pre‐developmental stages of fruit flavedo and albedo, which was a reason why the first three stages were harvested and processed in Huajuhong medicines (Figures S5 and S6). In addition, the PCA‐based MHLPs displayed first three stages were closer in both two HZYs (Figure S7a).

Furthermore, we constructed the transcriptome profile in 24 samples and approximately 493.13 Gb of clean data were filtered (Table S8). Subsequently, the fragments per kilobase of exon model per million mapped fragments (FPKM) of 26 924 genes were calculated (Table S9). Similar to the metabolome results, the PCA of the transcriptome also showed that the first three stages were closer in both two HZYs (Figure S7b). We removed the genes with a standard deviation = 0 in six developmental stages of flavedo or albedo. A total of 22 596 and 22 403 genes were filtered in flavedo and albedo, respectively (Table S10). Subsequently, we used the gap statistic (Nedyalkova *et al*., 2021) to determine the optimal number of clusters, and the numbers in flavedo and albedo were 7 and 8, respectively (Figure S8), which reflected that the two tissues have different regulatory network. Because flavedo was mainly source of Huajuhong medicines, we analysed the transcriptome regulatory network in flavedo and applied the fuzzy c‐means algorithm (Kumar and E Futschik, 2007) to cluster gene expression profiles in six stages of flavedo, and the seven distinct clusters of temporal patterns displayed different gene expression in flavedo (Figure 3b).

Among these clusters, cluster 1 represented gene expression that is upregulated then downregulated then upregulated, and stage 6 were highest, cluster 2 represented gene expression that was downregulated then upregulated, and stage 5 was lowest. Cluster 3 represented gene expression that is upregulated then downregulated, cluster 4 represented gene expression that is downregulated, cluster 5 represented gene expression that are upregulated, cluster 6 represented gene expression that is upregulated then downregulated, and stage 5 was highest. Cluster 7 represented gene expression that is upregulated then downregulated then upregulated, which displayed a bimodal expression pattern. Because flavones and flavanones were more than half of MHLPs, we analysed the expression of genes involved in flavone and flavanones pathway, *PAL* (*phenylalanine ammonia‐lyase*), *C4H*, *4CL* (*4‐coumarate‐‐CoA ligase*), *CHS*, *CHI*, *FNS* (*flavone synthase*), *F3′H* (*flavonoid 3′‐monooxygenase*) and *1,2RhaT* (1,2‐rhamnosyltransferase), which reflected that *PAL*, *CHS*, *CHI*, *F3′H* and *1,2RhaT* were downregulated, and *C4H*, *4CL* and *FNS* were upregulated then downregulated (Figure 3c), which further explained that pre‐developmental stages were harvested and processed time of Huajuhong, due to the high abundance of bioactive flavones and flavanones.

Subsequently, we used the MHLPs and seven clusters to perform co‐expression analysis, which reflected the potential regulatory networks of these metabolites (Figure 3d, Table S11). Among these relationships, 32 MHLPs were highly positively correlated with cluster 2, and 28 MHLPs were highly negatively correlated with cluster 5 (¦*r*¦ > 0.3). Therefore, the potential genes that regulated these MHLPs existed in clusters 2 and 5. For example, the flavonols, including 8‐C‐glucosyl‐5‐deoxykaempferol, quercetin 3‐sulphate and resokaempferol 7‐*O*‐hexoside were positively correlated with cluster 2, which included the R2R3‐MYB transcription factor that regulates the flavonol pathway (Liu *et al*., 2016).

### Identification of flavone regulatory genes

**Figure 4:**
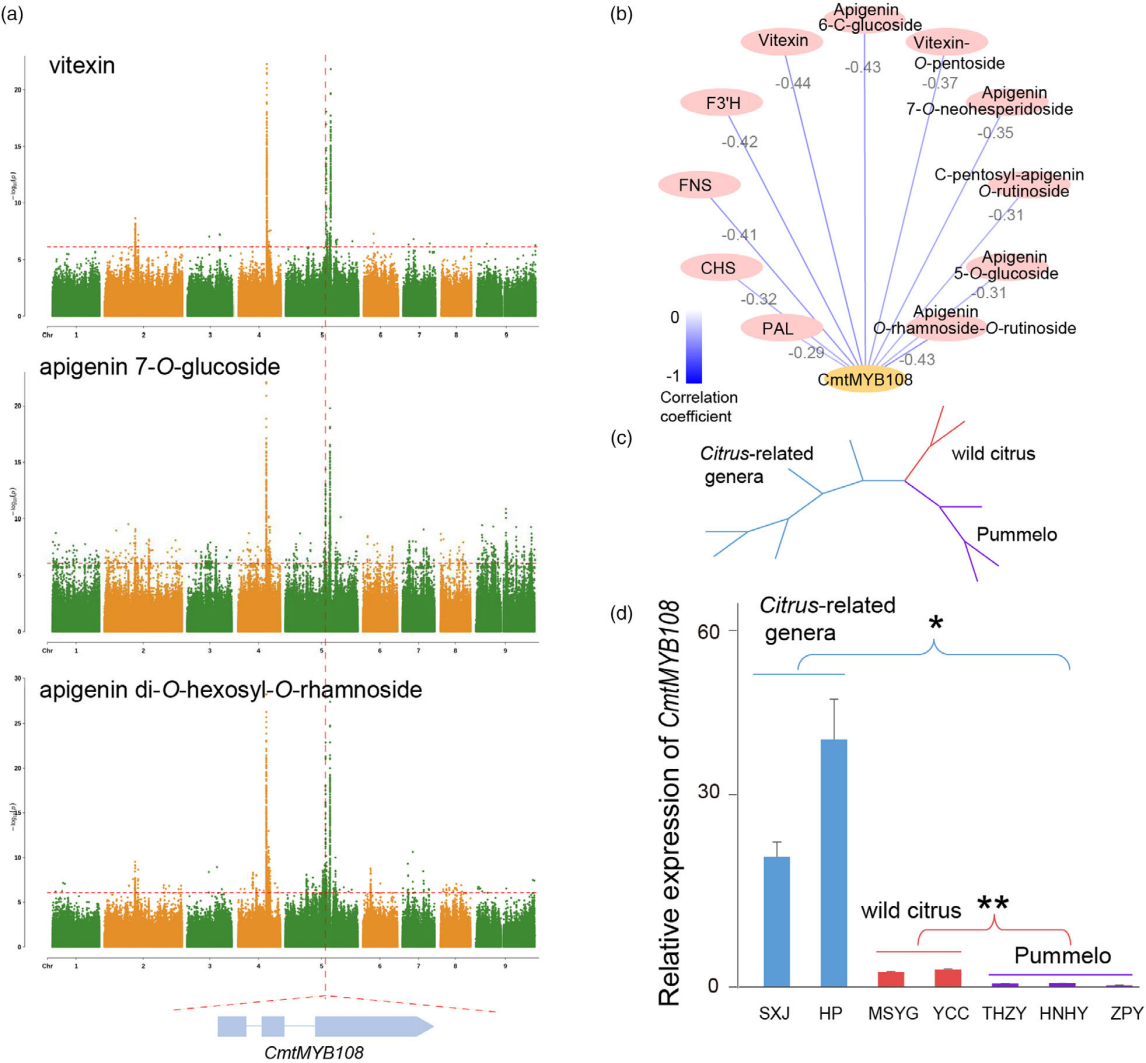
The identification and variation of *CmtMYB108*. (a) Manhattan plots of vitexin, apigenin 7‐*O*‐glucoside, apigenin di‐*O*‐hexosyl‐*O*‐rhamnoside. (b) Networks were established from correlations among flavones levels, expression of *CmtMYB108* and flavone biosynthesis pathway genes. Pearson correlation coefficient values were calculated for each pair, different line colour represents different correlation coefficient value, and grey number represent each pair correlation coefficient values. (c) Neighbour‐joining tree of *CmtMYB108* coding sequence of *Citrus*‐related genera, wild citrus species and pummelo. (d) Relative expression of *CmtMYB108* in the fruit peel of *Citrus*‐related genera (SXJ, *Glycosmis pentaphylla*. HP, *Clausena lansium*), wild citrus species (MSYG, *Citrus mangshanensis*. YCC, *Citrus ichangensis*) and pummelo (HZY‐T, *Citrus maxima* ‘Huazhouyou‐Tomentosa’. HNHY, *Citrus maxima* ‘Huanonghongyou’. ZPY, *Citrus maxima* ‘Zipiyou’).

Among the MHLPs, more than one‐third were flavones, such as vitexin, apigenin 7‐*O*‐glucoside, apigenin di‐*O*‐hexosyl‐*O*‐rhamnoside and apigenin 7‐*O*‐neohesperidoside, which are important bioactive metabolites in Huajuhong medicines (Figure S2a,b) (Mahmoud *et al*., 2019). To provide insight into the regulatory mechanism of flavones, we performed mGWAS in 154 pummelo accessions. Flavones, including vitexin, apigenin 7‐*O*‐glucoside and apigenin di‐*O*‐hexosyl‐*O*‐rhamnoside were co‐mapped to a significant SNP, located at Chr5:27438929 (Figure 4a, Tables S12–S14). The SNP is located 615 kb from *Cg5g022560* that is *Cmt069590* in HZY‐T. Cmt069590, named CmtMYB108, was grouped into the MYB transcription factor family that usually regulates the flavonoids pathway (Liu *et al*., 2015). Meanwhile, we found that *Cmt069590* grouped cluster 5, upregulated during the developmental stage, which was highly negative correlated with flavones in the MTA of the HZYs fruit flavedo (Figure 3d). We also found that gene expression was highly negative correlated with flavone biosynthesis pathway genes, including *PAL* (*Cmt048800*), *CHS* (*Cmt181890*), *FNS* (*Cmt079640*) and *F3′H* (*Cmt067750*), which were downregulated during the developmental stage (Figure 4b). Collectively, CmtMYB108 was a potential regulator of flavone pathway.

Due to the higher levels of flavones in pummelo than in *Citrus*‐related genera or wild citrus species, we analysed whether *CmtMYB108* was selected during the origin process of pummelo. The gene coding sequences in the citrus subfamilies were obviously divided into three groups, including *Citrus*‐related genera, wild citrus species and pummelo (Figure 4c). Meanwhile, we randomly selected two *Citrus*‐related genera accessions, two wild citrus accessions and three pummelo accessions to analyse the expression of *CmtMYB108*, which indicated that the expression of *CmtMYB108* was significantly lower in pummelo than in *Citrus*‐related genera/ wild citrus species (Figure 4d). Interestingly, we found a 0.6–0.9‐kb deletion ~3‐kb upstream of *CmtMYB108* existed in five *Citrus*‐related genera accessions, and an ~30‐bp insertion ~380‐bp upstream of *CmtMYB108* existed in five *Citrus*‐related genera and three wild citrus species (Figure S9). In addition, a miniature inverted‐repeat transposable element (MITE) and an MYC motif were found in the 0.6–0.9‐kb deletion of *Citrus*‐related genera, and an unknown motif was found in the ~30‐bp insertion (Figure S9). Collectively, the two SVs maybe explain the low expression of *CmtMYB108* in pummelo.

### CmtMYB108 Negatively regulates the flavone pathway

**Figure 5:**
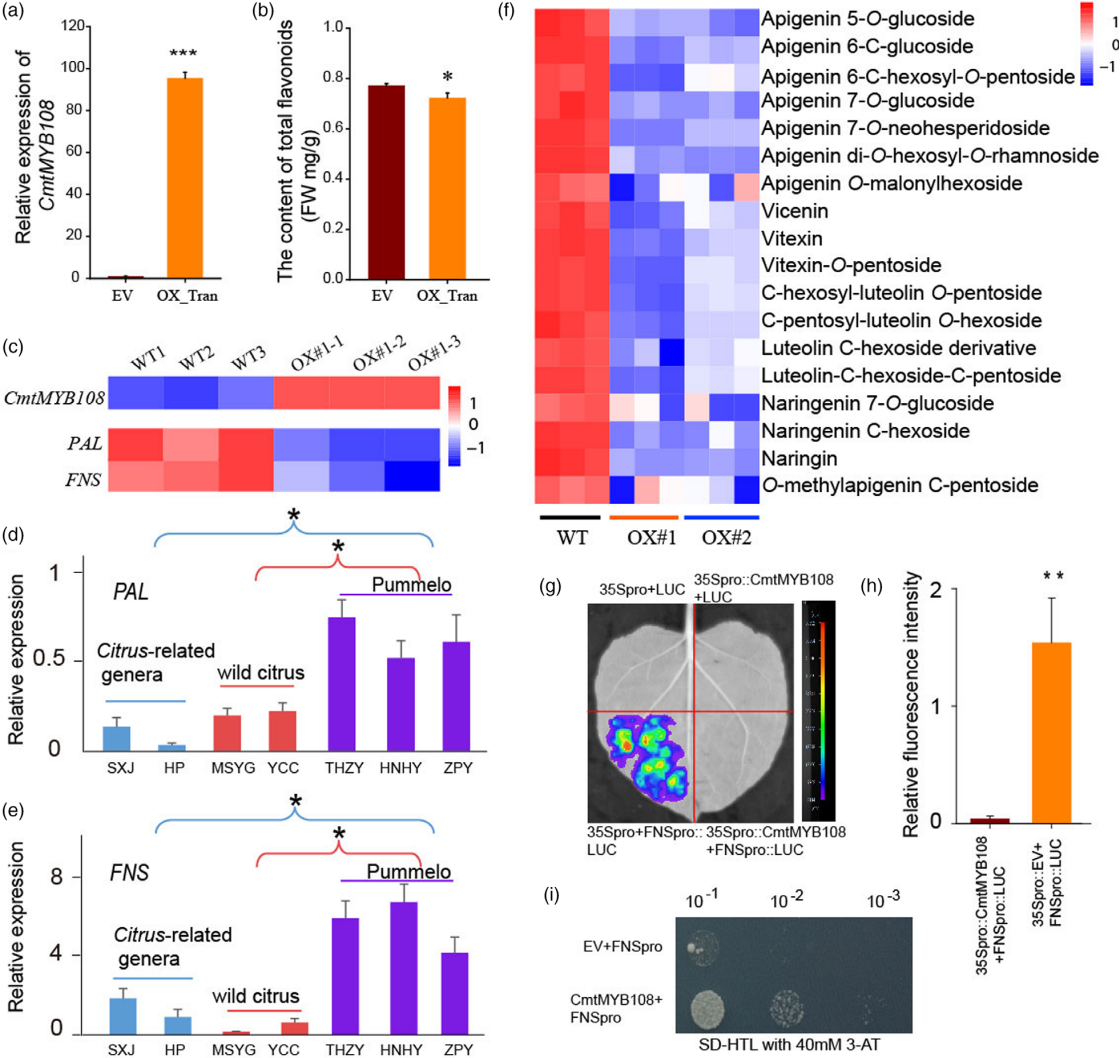
Functional analysis of *CmtMYB108*. (a) The relative expression of *CmtMYB108* in *N. benthamiana* leaves. EV: empty vector; OX Tran: *CmtMYB108* transiently overexpressed in *N. benthamiana* leaves. (b) The total content of flavonoids in *N. benthamiana* leaves. (c) The expression of *CmtMYB108*, *PAL* and *FNS* in sweet orange leaves. WT: wild type sweet orange; OX#1: *CmtMYB108* overexpressed in sweet orange. (d‐e) Relative expression of *CmtMYB108*, *PAL* and *FNS* in the fruit peel of *Citrus*‐related genera, wild citrus species and pummelo. (f) Heatmap showing that the content of flavones and flavanones significantly decreased in *CmtMYB108* overexpression lines. WT: wild type; OX#1: overexpressed *CmtMYB108*, OX#2: grafting line and the scion come from OX#1. (g) Transient transactivation assays in *N. benthamiana* leaves with firefly luciferase (LUC) reporter genes. (h) The relative fluorescence intensity in 35Spro::CmtMYB108+FNSpro::LUC and 35Spro::EV+FNSpro::LUC *N. benthamiana* leaves. (i) *CmtMYB108* directly bound to the promoter of *FNS* in the Y1H assay.

To further confirm the function of *CmtMYB108*, we transiently overexpressed it in *N. benthamiana* leaves, which suggested that the total content of flavonoids was significantly decreased in overexpressed *N. benthamiana* leaves (Figure 5a,b). Meanwhile, *CmtMYB108* was overexpressed in sweet orange by *Agrobacterium*‐mediated transformation. Metabolome analysis revealed that 15 flavones and 3 flavanones were significantly decreased in transgenic sweet orange (Figure 5f). These decreased flavones and flavanones are also consistent with the bioactive metabolites and their derivatives of MHLPs. Compared with wild‐type sweet orange leaves, the expression level of *CmtMYB108* was significantly increased, and the flavone pathway genes, *PAL* and *FNS* were significantly decreased in overexpressed sweet orange leaves (Figure 5c, Table S8). Interestingly, the expression levels of *PAL* and *FNS* were higher in pummelo compared with *Citrus*‐related genera/wild citrus species (Figure 5d,e), which is consistent with the accumulation of flavones and the expression levels of *CmtMYB108*.

To verify whether *CmtMYB108* negatively regulates the flavone pathway, the promoters of *FNS* and *PAL* were cloned for interaction analysis. A dual luciferase (LUC) transcriptional activity assay was performed in tobacco leaves, which confirmed that CmtMYB108 binds to the promoters of two genes and represses their expression levels (Figure 5g,h, Figure S10). Yeast one‐hybrid assays (Y1H) also revealed that CmtMYB108 interacted with the promoter of *FNS* (Figure 5i). Hence, these results revealed that CmtMYB108 is a negative regulator of flavone synthesis that functions by directly binding to the promoter of *FNS* and repressing its expression.

## Discussion

In this study, we profiled the changes in metabolite levels in the origin process of pummelo, which displayed that 59 metabolites (MHLPs) were significantly higher levels in pummelo. These MHLPs included 21 bioactive metabolites and 22 bioactive metabolite derivatives, accounting for 73% of the MHLPs number, and most bioactive metabolites and their derivatives were flavones, flavanones and limonoids, which is consistent with the metabolic basis of the medicinal value of Huajuhong medicines (Table S2). For example, vitexin and apigenin 7‐*O*‐neohesperidoside grouped flavones, and naringin grouped flavanones have anti‐inflammatory function, similar to previous studies (Cheng *et al*., 2017; Zhao *et al*., 2021) (Figure S2a,b), limonin and nomilin grouped limonoids were reported to have anti‐inflammatory, anti‐cancer, anti‐obesity (Fan *et al*., 2019; Sato, 2013). Therefore, the metabolic basis of pummelo medicines is consistent with most MHLPs.

HZYs were the most wide source of Huajuhong medicines. We assembled the HZY‐T genome with chromosome levels and high completeness (99.1%), which is better than the previous genome with contig levels and low completeness (94.1%) (Xian *et al*., 2022). Gene family analysis found that the expanded gene families in pummelo were enriched in the flavonoid biosynthetic process, phenylpropanoid biosynthetic process, response to oxidative stress and response to water deprivation. Pummelo was diverged approximately 7 Mya (Figure 2c) in the near northeastern India, northern Myanmar and northwestern Yunnan with high light, and gradually spread to southeast Asia and south of China (Wu et al., 2018; Yu *et al*., 2017). Due to the appearance of the quaternary glaciations, the climate has obviously become low temperature and drought after the time of pummelo divergence (Kirschner *et al*., 2022; Pedersen and Egholm, 2013). In previous studies, many plants enhanced their adaptability against UV‐B, low temperature and drought by increasing the levels of secondary metabolites, such as flavonoids and phenylpropanoid (Wang *et al*., 2021d; Zeng *et al*., 2020; Zhang *et al*., 2021). Pummelo may also adapt to changing environments with high light, low temperature and drought by accumulating high content of flavones and flavanones. Therefore, the origin processes of pummelo may indirectly promote the medicinal value formation of pummelo by enhancing its adaptability to harsh environment.

We found an R2R3 MYB transcription factor CmtMYB108 that repressed the flavone pathway and decreased the levels of flavones and flavanones, such as apigenine 7‐*O*‐neohesperidoside, vitexin, luteolin‐C‐hexoside‐C‐pentoside and naringin. Previous studies have been identified many transcription factor functions by MAT or mGWAS, including glycerophospholipid metabolism regulators in Rice Metabolic Regulation Network results (Yang *et al*., 2022), steroidal glycoalkaloids in the MicroTom Metabolic Network of tomato results (Li *et al*., 2020), and aromatic phenolamide biosynthesis regulators in the mGWAS results of Qingke (Zeng *et al*., 2020). Although the MAT of sweet orange has been reported (Feng *et al*., 2021), this study mainly focused on the accumulation mechanism of sucrose and acid that affected the fruit taste. Utilizing the MAT of HZY‐T and HZY‐S fruit flavedo, we systematically showed the regulatory network of bioactive metabolites in pummelo medicinal plants (Figure 3d, Table S11). In addition to identifying the new regulator network, we also confirmed the previously reported regulators that MYB42 increased the limonoids levels in *Citrus* (Zhang *et al*., 2020b). Collectively, multiomics analysis played an important role in determining the regulatory mechanism of bioactive metabolites in medicinal plants.

Transcription factors that regulate flavone biosynthesis are less known in plants compared with flavonol, anthocyanin and flavanol biosynthesis. Previous studies have shown that *GtMYBP3* and *GtMYBP4* in gentian flowers positively regulate flavone biosynthesis, while *CmMYB012* inhibits flavone biosynthesis in response to high temperatures in chrysanthemum (Nakatsuka *et al*., 2012; Zhou *et al*., 2021). In this study, multiomics analysis revealed that a novel R2R3 MYB transcription factor *CmtMYB108* potentially negatively regulated flavone biosynthesis. Overexpression of *CmtMYB108* in sweet orange significantly reduced the content of flavones in transgenic lines (Figure 5f). However, *CmtMYB108* overexpressed sweet orange showed growth defects with abnormal growth, dwarfing and minimal leaves. Only one transgenic seedling with medium expression level of *CmtMYB108* survived, which might be due to the inhibition of flavones, the essential metabolites for plant growth and development (Morales‐Quintana and Ramos, 2021). In addition, previous studies reported that overexpression of *AtMYB62*, homologous gene of *CmtMYB108* in *Arabidopsis*, also led to abnormal development, dwarfing and growth retardation in *Arabidopsis* (Devaiah *et al*., 2009). Further detection found that the expression levels of the flavone pathway genes *PAL* and *FNS* were significantly upregulated in pummelo compared with *Citrus*‐related genera and wild citrus species (Figure 5d,e). Through the LUC assay, we found that CmtMYB108 inhibited the promoter activities of *PAL* and *FNS*, and the Y1H experiment showed that only the *FNS* promoter could be bound by CmtMYB108 (Figure 5g–i, Figure S10). The above results indicated that CmtMYB108 could directly bind and inhibit the expression of *FNS*, thereby inhibiting the synthesis of flavones. MITEs are short non‐autonomous DNA transposons, that are widely studied in plants and found to exist in promoters or other regulatory regions to play important roles in gene expression regulation (Mao *et al*., 2015; Shen *et al*., 2017; Wang *et al*., 2021a; Zheng *et al*., 2019). Here, two SVs, including MITE, MYC motif and unknown motif, were observed in the promoter region of *CmtMYB108* in *Citrus*‐related genera and wild citrus species (Figure S9), which may decrease the expression of *CmtMYB108* in pummelo, indirectly increase expression of *PAL* and *FNS* and result in a higher content of flavones in pummelo.

In conclusion, our study elucidates the formation of bioactive flavones during the origin process of pummelo. HZY‐T was regarded as a representative to explain the changes in the genome during origin process of pummelo. We also constructed MHLPs transcriptional regulation networks. Furthermore, a novel R2R3 MYB transcription factor, CmtMYB108, was identified by multiomics analysis to regulate the synthesis of flavones by directly inhibiting the promoter activity of *FNS*. In addition, the two SVs in the promoter region of *CmtMYB108* were identified, which maybe result in a decrease in the expression of *CmtMYB108* in pummelo, and promote the accumulation of flavones. This study provides a new reference for the improvement and breeding of medicinal citrus in the future.

## Methods

### Plant materials

The 72 samples (three biology replicates) of HZY‐T and HZY‐S were from Huazhou, Guangdong province from April to September 2021. Five to 10 fruit were randomly divided into three replicates. The fruits were washed with tap water, then the flavedo and albedo were separated and placed in liquid nitrogen followed by storage at −80 °C. The 22 accessions fruit peel and flesh for metabolism analysis were collected from Yunnan province, Guangxi province and Hubei province in the years of 2019 and 2020 (Table S1, https://doi.org/10.6084/m9.figshare.22261738). The fruit samples were ripe, a normal size and healthy. Nine to 15 fruit were randomly divided into three replicates. Each piece of fruit was washed with tap water. The flesh was separated and placed in liquid nitrogen followed by storage at −80 °C until further analysis.

### Metabolite profiling

All the chemicals were of analytical reagent grade. Gradient‐grade methanol, acetonitrile and acetic acid were purchased from Merck Company, Germany. The water was doubly deionized with Milli‐Q water purification system (Millipore, Bedford, MA). Standards were purchased from ANPEL, Shanghai, China, BioBioPha Co., Ltd. and Sigma‐Aldrich, USA.

The freeze‐dried fruits were crushed using a mixer mill (MM 400, Retsch) with zirconia beads for 1.8 min at 15 Hz. A 100 mg mass of powder was weighted and extracted overnight at 4 °C with 1.0 mL of 70% aqueous methanol. Following centrifugation at 10 000 ***g*** for 10 min, the extracts were filtered (SCAA‐104, 0.22 μm pore size; ANPEL, Shanghai, China) before LC–MS analysis. The sample extracts were analysed using an LC‐ESI‐MS/MS system (Shim‐pack UFLC SHIMADZU CBM30A system, http://www.shimadzu.com.cn/; MS, SHIMADZU LCMS‐8060, http://www.shimadzu.com.cn/). The analytical conditions were as follows, UPLC: column, Shim‐pack GISS C18 (pore size 1.9 μm, dimensions 2.1 × 100 mm); solvent system, water (0.04% acetic acid): acetonitrile (0.04% acetic acid); gradient program, 95:5 V/V at 0 min, 5:95 V/V at 12.0 min, 5:95 V/V at 13.2 min, 95:5 V/V at 13.3 min, 95:5 V/V at 15.0 min; flow rate, 0.4 mL/min; temperature, 40 °C; and injection volume: 2 μL.

Population structure analyses by metabolomics principal component analysis plots were used to infer the structure of the *Citrus*‐related genera, wild citrus species and pummelo. The data matrix was generated from *Citrus*‐related genera, wild citrus species and pummelo with 403 metabolites which represented the contents of each metabolite in average of two biological repeats. PCA was performed with log2‐transformed metabolite data. PCA was performed with FactoMineR and factoextra packages in R version 3.6.2. Significantly difference analysis was performed with wilcox.test in R version 3.6.2.

### The anti‐inflammation function identification of vitexin, apigenin 7‐*O*‐neohesperidoside and naringin

RAW 264.7 macrophages were grown in Dulbecco's Modified Eagle's Medium (DMEM) with high glucose (4.5 g/L) (Hyclone, GE Healthcare, Little Chalfont, UK) containing 10% fetal bovine serum (FBS) supplemented with 1% penicillin and streptomycin at 37 °C and 5% CO2–95% air under humidified conditions. The concentrations of vitexin, apigenin 7‐*O*‐neohesperidoside and naringin were 3 μmol/L. In brief, the RAW 264.7 macrophages were routinely cultured in a 12‐well for 24 h. Then, the cells were co‐treated with flavones or flavanones (3 μmol/L) and LPS (1 μg/mL) for additional 18 h under cell culture conditions.

### Library construction and sequencing

HZY‐T sample for genome assembly and HZY‐S were re‐sequenced were selected from Huazhou, Guangdong province. Extraction of genomic DNA from leaf tissue using TIANGEN BIOTECH (BEIJING) DNAquick Plant System from HZY‐T and HZY‐S, respectively. The 150‐bp paired‐end libraries of HZY‐T and HZY‐S were then constructed using the Illumina Genomic DNA Sample Preparation Kit, and sequencing was performed using Illumina NovaSeq 6000 platforms. For PacBio long‐read sequencing, we use the protocol then released by PacBio to construct the SMRTbell libraries (20 kb) of HZY‐T, then use Pacbio Sequel platform II for sequencing. A total of 7 995 694 (~100×) Pacbio subreads were obtained. In addition, an Hi‐C libraries were created from tender leaves of HZY‐T by Novogene (Beijing, China), A total of 92.1 million (~100×) 150 bp paired‐end reads were produced on the Illumina NovaSeq 6000 platform.

### Genome assembly

The HZY‐T genome size is first estimated using GCE (v1.0.2) (Liu *et al*., 2013). Then use Canu (v2.0) (Koren *et al*., 2017) to correct (parameter ‘maxThreads = 20, minReadLength = 2000, minOverlapLength = 500, corOutCoverage = 150, corMinCoverage = 2’), trim (parameter ‘maxThreads = 20 minReadLength = 2000, minOverlapLength = 500’) and assemble (parameter’ maxThreads = 25, genomeSize = 363 m, correctedErrorRate =0.035′). The PacBio subreads to obtain a diploid HZY‐T genome.

In order to obtain the HZY‐T haploid genome, we first used Minimap2 (Li, 2018) to map the trimmed Pacbio subreads to the initial diploid genome. Then use purge_dups (Guan *et al*., 2020) to remove redundancy and get the main haploid assembly. Finally, the Nextpolish (Hu *et al*., 2020) was used to polish the haploid assembly with a short read‐long sequence and rimmed Pacbio subreads. After finishing these steps, a preliminary evaluation of the quality of contigs by assembled size, N50, longest sequence was undertaken. BUSCO (Manni *et al*., 2021) was used to evaluate the completeness.

For HZY‐T pseudochromosome construction, we first mapped the clean Hi‐C reads to the polished assembly using BWA. Then, the contigs is anchored to scaffolds using ALLHiC (parameter ‘‐e AAGCTT‐k 10’). We finally aligned the ALLHiC (Zhang *et al*., 2019) assembly against the pummelo genome (Citrus grandis (L.) Osbeck.cv. ‘Wanbaiyou’ v1.0) using NUCmer in MUMMER4 (Marcais *et al*., 2018) with default parameters to determine the pseudochromosome order.

### Genome mapping, variant calling and population analyses

Raw Illumina reads was processed to remove adapter sequences and low‐quality reads by Fastp (Chen *et al*., 2018). The cleaned reads were mapped to the reference genome using BWA‐MEM (Li and Durbin, 2009). Then mapped reads were sorted and the duplicated reads were removed by Sortbam and MarkDuplicates tools in the GATK package (McKenna *et al*., 2010). The UnifiedGenotyper of GATK was then used to call variants. The fourfold synonymous third‐codon transversion (4DTV) file was extracted in VCF file by SnpEff (Cingolani *et al*., 2012). The PCA was performed by PLINK (Purcell *et al*., 2007) and GCTA (Yang *et al*., 2011) using 4DTV file.

### Gene family analysis and phylogenetic tree

The longest proteins of 11 genomes were filtered. The gene families were identified by OrthoFinder (Emms and Kelly, 2019). The gene family's number of each genome was computed by CAFÉ (De Bie *et al*., 2006). For Phylogenetic tree analysis, we used MUSCLE (v3.8.31) (Edgar, 2004) to multiple sequence alignment. The conserved sequences were extracted and merged by Gblocks_0.91b (Castresana, 2000) and SeqKit (Shen *et al*., 2016), respectively. Then we used RAxML (Stamatakis, 2014) to construct the ML tree.

### Transposable elements and genes annotation for HZY‐T

The genome sequences were used to build a *de novo* TE library using the RepeatModeler software (Flynn *et al*., 2020). The TE library was used to identify repeat sequences in particular genomes using RepeatMasker. Gene models were annotated based on ab initio gene predictions, homology searches and RNA‐seq. For ab initio gene predictions, AUGUSTUS (Nachtweide and Stanke, 2019), GlimmerHMM (Majoros *et al*., 2004) and SNAP (Korf, 2004) were employed using default parameters. The protein databases were constructed by integrating the amino acid sequences from the published genomic protein sequences of Citrus. Homology searching was then conducted using genome threader. In addition, RNA‐seq reads were generated from a mixture of tissues. The Trinity software was utilized to perform genome‐guided and de novo transcript assembly. The PASA (Avram *et al*., 2021) software was used to update the protein‐coding gene annotations by incorporating PASA alignment evidence, correcting exon boundaries, adding UTRs and modelling alternative splicing based on the PASA alignment assemblies. All of the gene structures predicted using the aforementioned methods were combined using the EVM software (Haas *et al*., 2008).

### Structural variation analysis

The longest 30× PacBio/Nanopore reads were mapped to the reference by NGLMR (Sedlazeck *et al*., 2018). The resulting alignments were sorted and indexed by Samtools (Li *et al*., 2009). Initial SV callings were performed by Sniffles, SVs supported by at least five reads were left. We filtered low‐quality SVs (flag: UNRESOLVED) and removed duplicate SV calls (SVs at the same position for multiple pairs of breakpoints). Next, we merged SVs from all individuals using SVRVIVOR (Jeffares *et al*., 2017) with parameters “200 ‐1 1 ‐1 ‐1 ‐1 merged.vcf”. The merged SVs were used as input to force call all the SVs across all samples using Sniffles with parameter –Ivcf enabled. Finally, we merged the called SVs again to obtain a fully genotyped multi‐sample SVs. The merged SVs were added to the genome of *C. sinensis* to construct a graph‐based genome with the vg pipeline (Hickey *et al*., 2020).

### Transcriptome analysis

Raw Illumina reads were processed to remove adapter sequences and low‐quality reads by Fastp (Chen *et al*., 2018). The cleaned reads were mapped to the reference genome using HISAT2 (Kim *et al*., 2019). Then mapped reads were sorted by Samtools (Li *et al*., 2009). FPKM values were calculated by Subreads (Liao *et al*., 2014) in R software.

### Determination of total flavonoid content

The total flavonoid content of tobacco leaf was measured with an aluminium chloride method. Briefly, 0.5 g fresh leaf was powdered and extracted with 10 mL 80% methanol, shaking at room temperature for 2 h, and centrifuged at 1400 ***g*** to get the supernatant. Then prepare the reaction according to the following steps: 0.5 mL supernatant, 2.25 mL ddH_2_O and 0.15 mL 5% NaNO_2_ were mixed and shaken for 6 min, then added 0.3 mL 10% AlCl_3_ solution, shaken for 5 min, finally added 1 mL 1 M NaOH solution and immediately measured the absorbance at 510 nm with a spectrophotometer. Rutin was used as the standard curve to calculate the content of total flavonoids.

### Plasmid construction and stable transformation in citrus

The coding sequence of CmtMYB108 was isolated from HZY‐T pummelo by PCR and cloned into a pK7WG2D overexpression vector. The vector was then transformed into epicotyls of Anliu sweet orange by using A. tumefaciens strain EHA105 described previously (Hao *et al*., 2016). The explants were screened by GFP and then the expression levels of CmtMYB108 were identified by qPCR. The positive transgenic seed lines were potted in a controlled greenhouse for subsequent studies.

### Dual luciferase transcriptional activity assay

About 2 kb of DNA sequences upstream of the translational start codon of *FNS* (*Cmt079640*) and *PAL* (*Cmt241630*) were amplified by PCR from genomic DNA of ‘Anliu’ sweet orange. The fragments were subsequently inserted into a pGreenII 0800‐LUC to generate reporter vectors, which were then transformed into *A. tumefaciens* GV3101 (with plasmid pSoup‐p19) competent cells. The effector vector was a CmtMYB108 overexpression vector pK7WG2D described above, and an empty pK7WG2D vector was used as a control. Both vectors were also transformed into GV3101 (pSoup‐p19) competent cells. The GV3101 cells containing effector and reporters were mixed to a proportion of 5:1 and then injected into leaves of *N. benthamiana*. 3 days after injection, the surface of the transfected leaves was treated with 0.2 mM luciferin and kept for 5 min in darkness. LUC activity was measured using a NIGHTSHADE imaging apparatus (LC 985). The primers used for these experiments are listed in Table S15.

### Yeast one‐hybrid assay analysis

The promoters of *CsFNS* and *CsPAL* were ligated into the pHIS2 vector (Clontech) which contains a HIS3 nutritional reporter gene. The bait plasmids were then integrated into a yeast strain Y187. 3‐AT (3‐amino‐1,2,4‐triazole) was used for inhibiting the self‐activation of the bait vectors. Full length of CmtMYB108 was ligated into the pGADT7 vector (Clontech) and then transferred into yeast cells containing bait vectors. pGADT7 empty vector used as negative control. The positive interactions could be detected by the growth of yeast cells on histidine‐deficient media.

### RNA extraction and gene expression analysis

RNA extractions from all frozen samples were performed as described in a previous study (Liu *et al*., 2006). Then, 1.0 mg of the extract was digested with 4× gDNA wiper (Vazyme Biotech) to remove the genomic DNA, followed by the addition of 5× HiScriptII Q RT supermix to synthesize first‐strand cDNA for further analysis. The relative expression of candidate genes, including *CmtMYB108* and its target genes (*PAL* and *FNS*), was quantified using quantitative RT‐PCR with the SYBR FAST qPCR Kit (YEASEN) and the LC480 Fast Real Time System. qRT‐PCR was performed using gene‐specific primers (Table S15) and equal amounts of cDNA from three independent biological replicates with three technical replicates for each biological replicate. Relative expression levels were calculated using the 2^−ΔΔCt^ method.

## Declaration of interests

The authors declare no competing interests.

## Author contribution

Z.M., (Zhaocheng Ma), L.H. and Q.X. conceived the project and supervised this study. W.Z., (Weikang Zheng) performed genome, transcriptome and metabolome analysis. W.Z., (Wang Zhang) performed the gene function identification. M.Y. and Y.H. performed genome assembled. S.L. performed genome annotation. X.W., S.W. and S.S. performed mGWAS analysis. Z.M., (Zhaocheng Ma). coordinated the project with help from X.D., J.L., L.G., Z.M. (Zhinan Mei) and D.L. W.Z., (Weikang Zheng) and W.Z., (Wang Zhang) wrote the manuscript with contributions from Y.X., S.Y., B.H., T.Y. W.Z., (Weikang Zheng), X.L., Q.Z. and L.Y., performed anti‐inflammation experiment.

## Supporting information


**Figure S1** The PCA of fleshes (a) and peels (b) of 22 accessions based on 403 metabolites levels.
**Figure S2** Effect of vitexin, naringin and apigenin 7‐*O*‐neohesperidoside on LPS‐induced mRNA expression of *IL‐6* (a) and *COX‐2* (b) in RAW 264.7 macrophages.
**Figure S3** Heatmap showing Hi‐C interactions of HZY‐T.
**Figure S4** Phylogenetic tree of the 22 pummelo accessions. The tree was constructed by maximum likelihood tree.
**Figure S5** The heatmap of 59 MHLPs in 12 HZY‐T and HZY‐S fruits flavedo samples.
**Figure S6** The heatmap of 59 MHLPs in 12 HZY‐T and HZY‐S fruits albedo samples.
**Figure S7** (a) PCA results for the MHLPs data from 24 HZY‐T and HZY‐S samples.
**Figure S8** The optimal number of clusters in six stages transcriptome data of flavedo (a) and albedo (b).
**Figure S9** Diagram of *CmtMYB108* promoter sequence variations in Aurantioideae species.
**Figure S10** Transient transactivation assays in *N. benthamiana* leaves with firefly luciferase (Luc) reporter genes.

**Table S1** Accession used for metabolome analysis.
**Table S2** Average of two biological replicates of 403 metabolites in 22 accessions.
**Table S3** The genome information of THZY.
**Table S4** Statistics of genome sequence data of Citrus accessions used in this study.
**Table S5** The constraction and expansion gene families in pummelo compared with the Expansion in pummelo compared with the Citrus‐related genera or wild citrus.
**Table S6** The GO enrichment results of expansion gene fimalies in pummelo compared with Citrus‐related genera and wild citrus.
**Table S7** The genes with structural variations.
**Table S8** The RNA‐seq information of 24 HZY‐T and HZY‐S samples.
**Table S9** The gene average FPKM of three replicates in albedo and albedo of THZY and SHZY.
**Table S10** The genes with the standard deviation > 0.
**Table S11** The regulation network of positively selective metabolites in flavode.
**Table S12** mGWAS results of vitexin.
**Table S13** mGWAS results of apigenin 7‐O‐glucoside.
**Table S14** mGWAS results of apigenin di‐O‐hexosyl‐O‐rhamnoside.
**Table S15** The primers of genes were used in this study.

## Data Availability

HZY‐T genome assembly data in this study have been deposited at DDBJ/ENA/GenBank under BioProject ID PRJNA911419. The resequencing data were listed in Table S4. The accession numbers of RNA‐seq data for genes annotation was SRR22744601.
