# Supplementary material for: Evolution‐guided multiomics provide insights into the strengthening of bioactive flavone biosynthesis in medicinal pummelo
Source: Plant Biotechnol J. 2023 Apr 28;21(8):1577–89. doi: 10.1111/pbi.14058 (PMC10363765; doi:10.1111/pbi.14058)
Supplement: Supplementary file 1 — Figure S1 The PCA of fleshes (a) and peels (b) of 22 accessions based on 403 metabolites levels. Figure S2 Effect of vitexin, naringin and apigenin 7‐O‐neohesperidoside on LPS‐induced mRNA expression of IL‐6 (a) and COX‐2 (b) in RAW 264.7 macrophages. Figure S3 Heatmap showing Hi‐C interactions of HZY‐T. Figure S4 Phylogenetic tree of the 22 pummelo accessions. The tree was constructed by maximum likelihood tree. Figure S5 The heatmap of 59 MHLPs in 12 HZY‐T and HZY‐S fruits flavedo samples. Figure S6 The heatmap of 59 MHLPs in 12 HZY‐T and HZY‐S fruits albedo samples. Figure S7 (a) PCA results for the MHLPs data from 24 HZY‐T and HZY‐S samples. Figure S8 The optimal number of clusters in six stages transcriptome data of flavedo (a) and albedo (b). Figure S9 Diagram of CmtMYB108 promoter sequence variations in Aurantioideae species. Figure S10 Transient transactivation assays in N. benthamiana leaves with firefly luciferase (Luc) reporter genes. [file PBI-21-1577-s002.docx]

**Supporting Information**


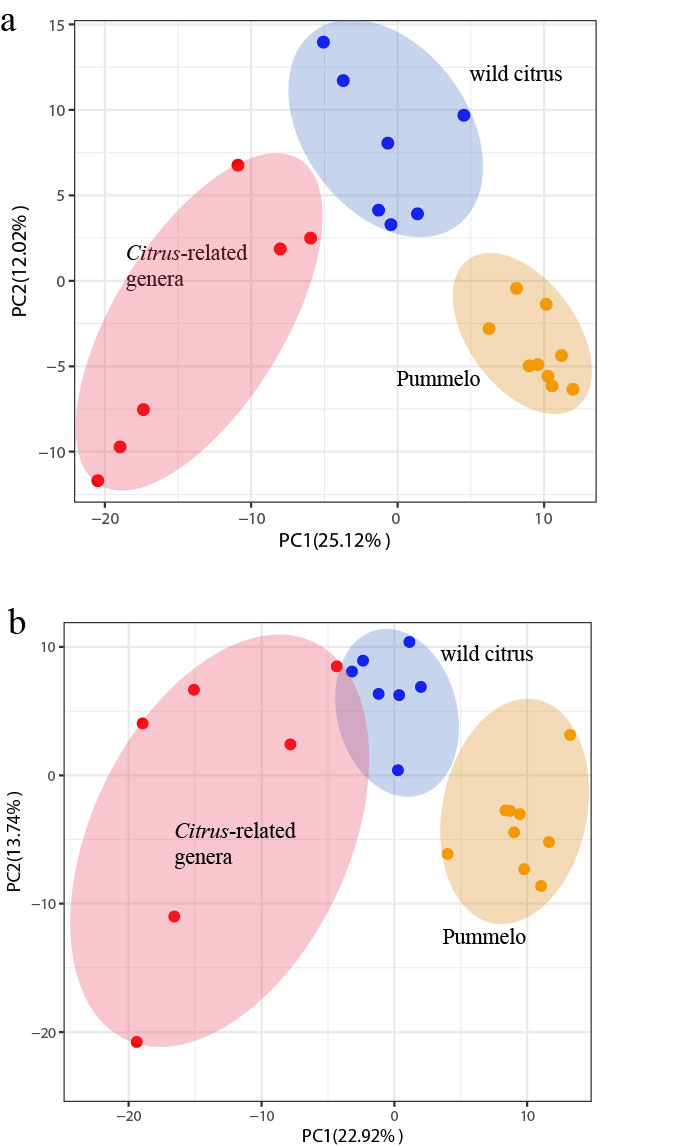


**Figure S1 The PCA of fleshes (a) and peels (b) of 22 accessions based on 403 metabolites levels.**


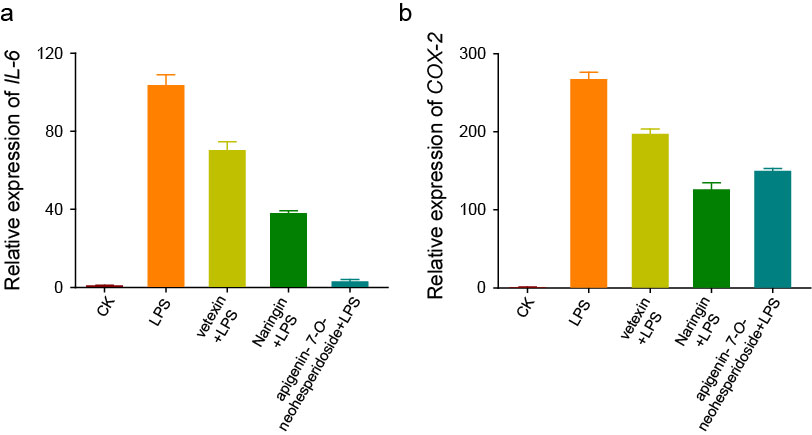


**Figure S2 Effect of vitexin, naringin and apigenin 7-*O*-neohesperidoside on LPS-induced mRNA expression of *IL-6* (a) and *COX-2* (b) in RAW 264.7 macrophages. The concentration of LPS is 1 μg/mL. The concentration of vitexin, naringin and apigenin 7-*O*-neohesperidoside is 3 μmol/L.**


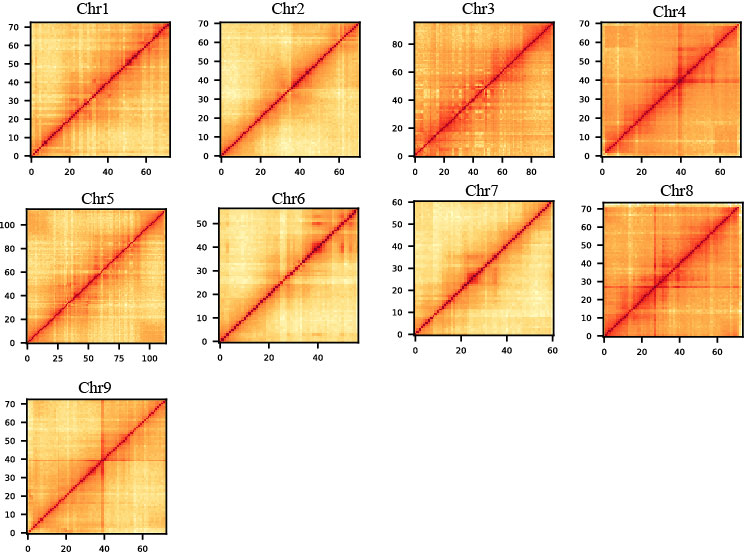


**Figure S3 Heatmap showing Hi-C interactions of HZY-T.**


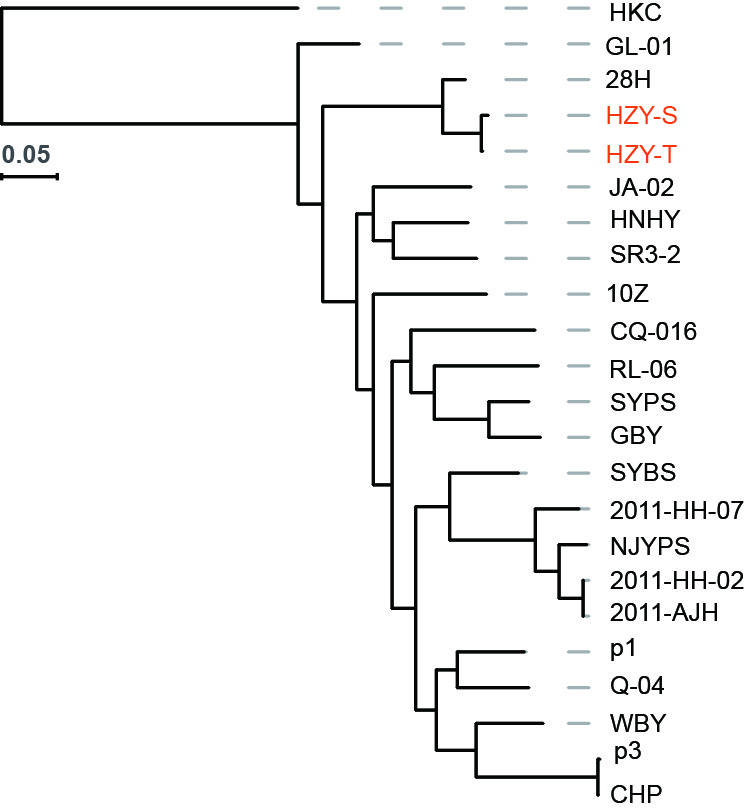


**Figure S4 Phylogenetic tree of the 22 pummelo accessions. The tree was constructed by maximum likelihood tree.**


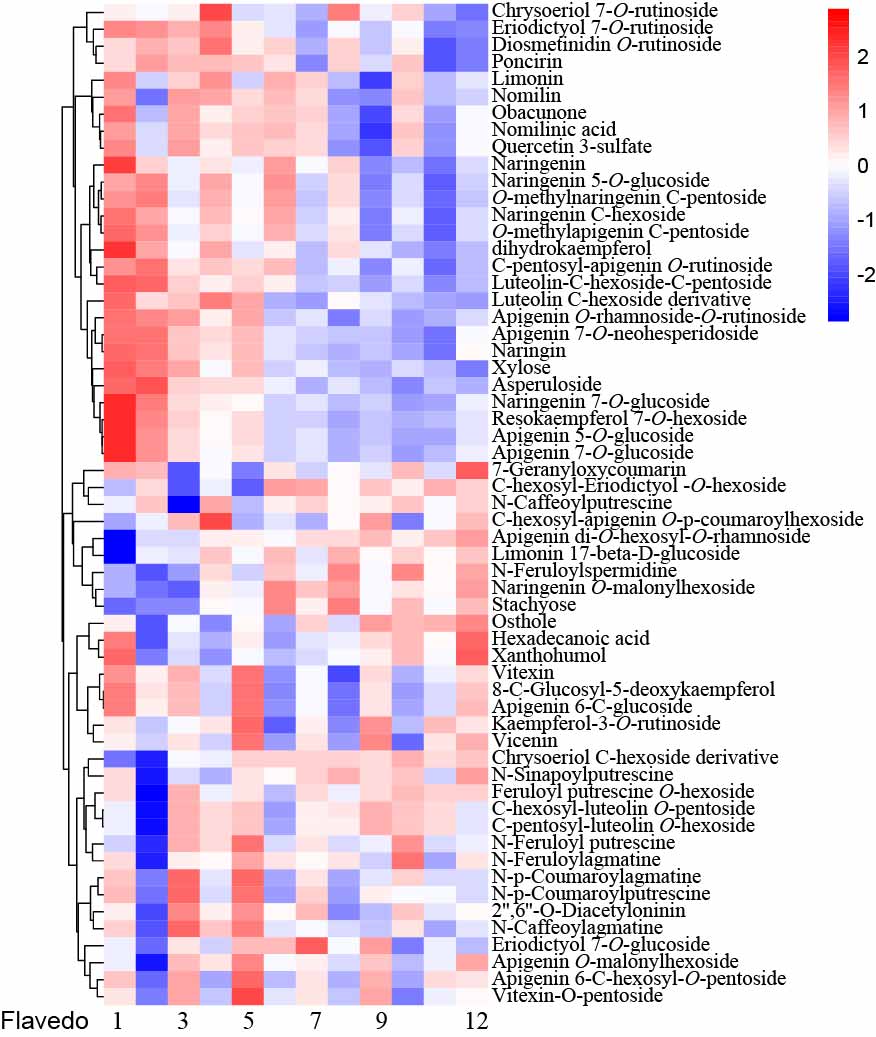


**Figure S5 The heatmap of 59 MHLPs in 12 HZY-T and HZY-S fruits flavedo samples.**


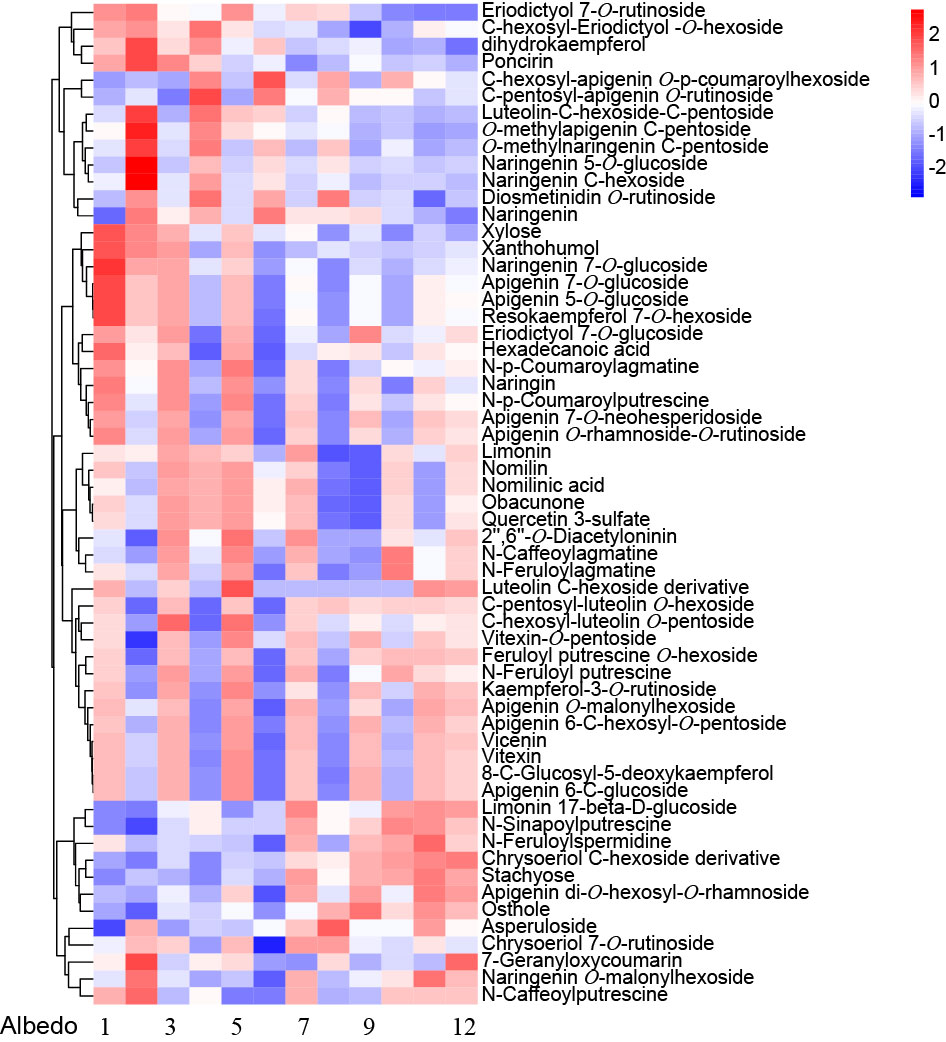


**Figure S6 The heatmap of 59 MHLPs in 12 HZY-T and HZY-S fruits albedo samples.**


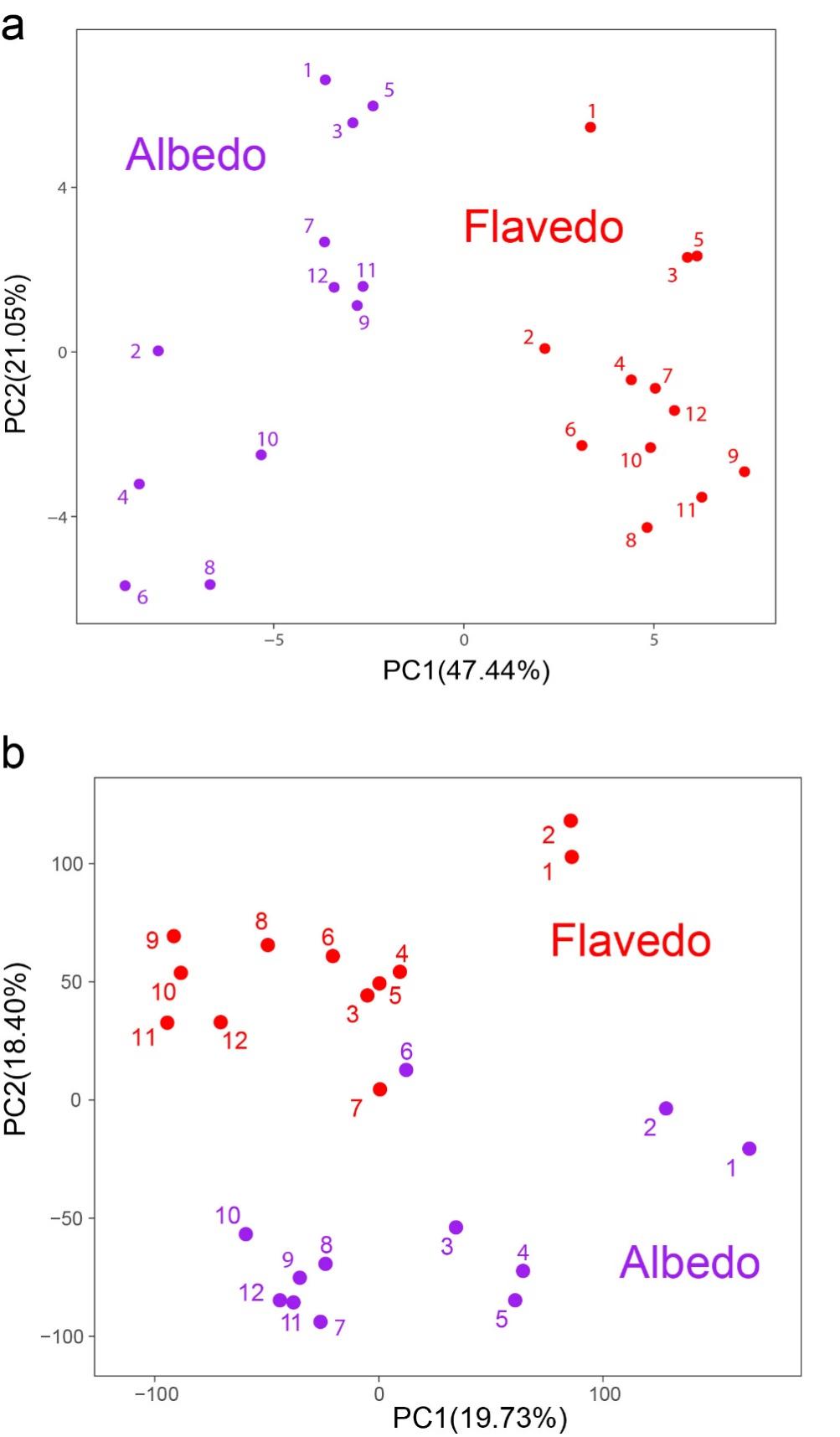


**Figure S7 (a) PCA results for the MHLPs data from 24 HZY-T and HZY-S samples. Purple dots represent fruit albedo tissues. Red dots represent fruit flavedo tissues.** **1, 3, 5, 7, 9, 11 represent stage 1 to 6 of HZY-T fruits. 2, 4, 6, 8, 10 ,12 represent stage 1 to 6 of HZY-S fruits. (b) PCA results for the transcriptome data from 24 HZY-T and HZY-S samples. Purple dots represent fruit albedo tissues. Red dots represent fruit flavedo tissues. 1, 3, 5, 7, 9, 11 represent stage 1 to 6 of HZY-T fruits. 2, 4, 6, 8, 10 ,12 represent stage 1 to 6 of HZY-S fruits.**


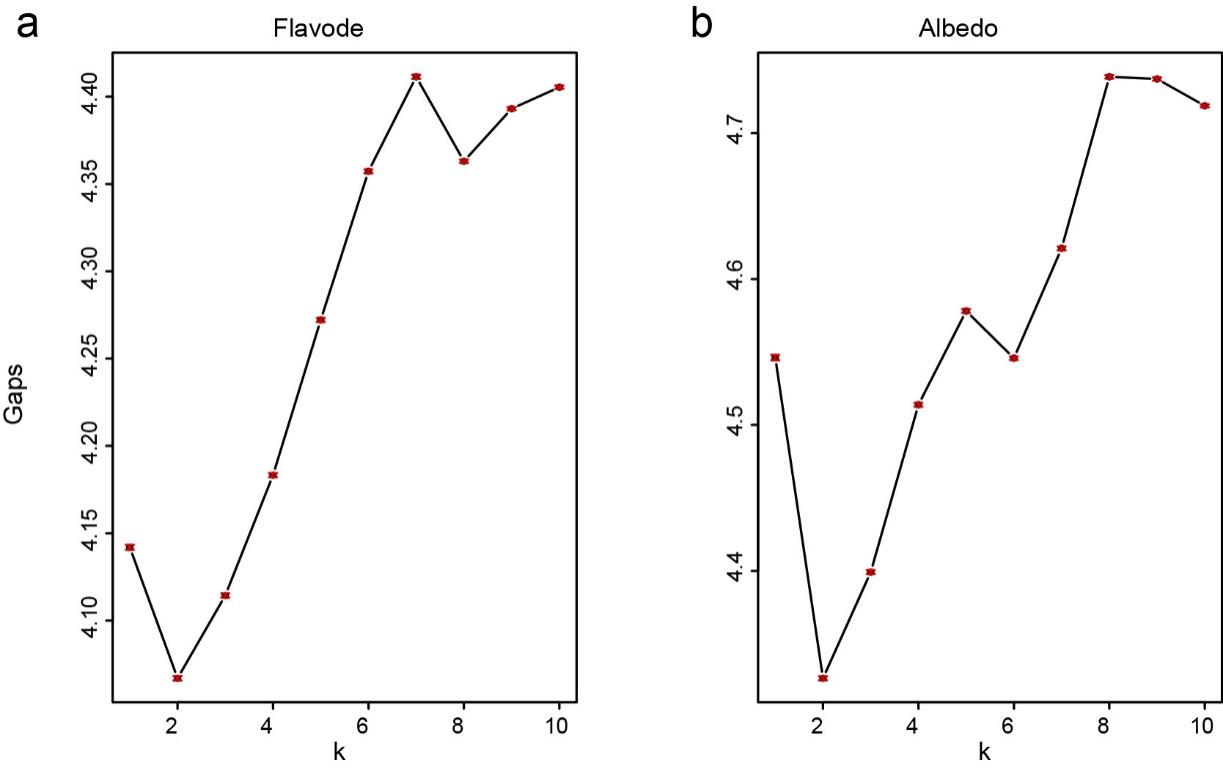


**Figure S8 The optimal number of clusters in six stages transcriptome data of flavedo (a) and albedo (b).**


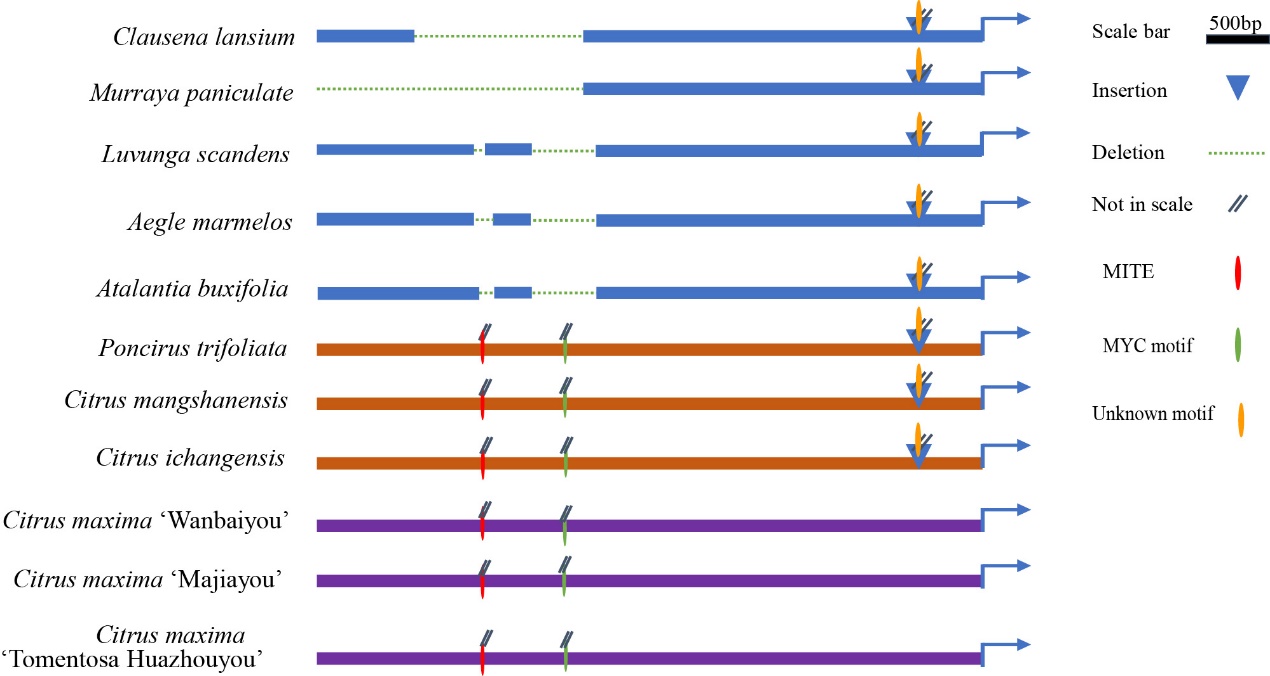


**Figure S9 Diagram of *CmtMYB108* promoter sequence variations in Aurantioideae species. The sequence from HZY-T was used as reference. The hooked arrow marks the transcription start sites.**


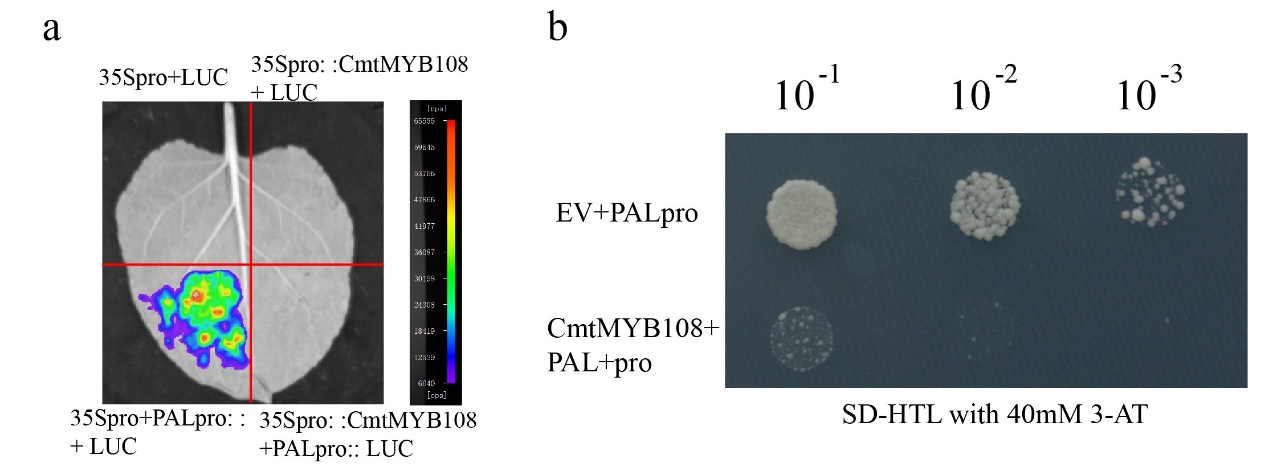


**Figure S10 Transient transactivation assays in *N. benthamiana* leaves with firefly luciferase (Luc) reporter genes. The promotor of *PAL* from sweet orange was cloned into pGreen0800 LUC vector to drive Luc.**
